# Telemedicine in Intensive Care Units: Protocol for a Scoping Review

**DOI:** 10.2196/19695

**Published:** 2020-12-31

**Authors:** Camille Guinemer, Martin Boeker, Bjoern Weiss, Daniel Fuerstenau, Felix Balzer, Akira-Sebastian Poncette

**Affiliations:** 1 Department of Anesthesiology and Intensive Care Medicine Charité-Universitätsmedizin Berlin Corporate member of Freie Universität Berlin, Humboldt-Universität zu Berlin, and Berlin Institute of Health Berlin Germany; 2 Faculty of Medicine Institute of Medical Biometry and Statistics University of Freiburg Freiburg Germany; 3 Copenhagen Business School Copenhagen Denmark; 4 School of Business & Economics Freie Universität Berlin Berlin Germany; 5 Einstein Center Digital Future Berlin Germany

**Keywords:** tele-ICU, intensive care unit, intensive care, telemedicine, critical care, implementation, scoping review

## Abstract

**Background:**

Telemedicine has been deployed to address issues in intensive care delivery, as well as to improve outcome and quality of care. Implementation of this technology has been characterized by high variability. Tele-intensive care unit (ICU) interventions involve the combination of multiple technological and organizational components, as well as interconnections of key stakeholders inside the hospital organization. The extensive literature on the benefits of tele-ICUs has been characterized as heterogeneous. On one hand, positive clinical and economical outcomes have been shown in multiple studies. On the other hand, no tangible benefits could be detected in several cases. This could be due to the diverse forms of organizations and the fact that tele-ICU interventions are complex to evaluate. The implementation context of tele-ICUs has been shown to play an important role in the success of the technology. The benefits derived from tele-ICUs depend on the organization where it is deployed and how the telemedicine systems are applied. There is therefore value in analyzing the benefits of tele-ICUs in relation to the characteristics of the organization where it is deployed. To date, research on the topic has not provided a comprehensive overview of literature taking both the technology setup and implementation context into account.

**Objective:**

We present a protocol for a scoping review of the literature on telemedicine in the ICU and its benefits in intensive care. The purpose of this review is to map out evidence about telemedicine in critical care in light of the implementation context. This review could represent a valuable contribution to support the development of tele-ICU technologies and offer perspectives on possible configurations, based on the implementation context and use case.

**Methods:**

We have followed the Preferred Reporting Items for Systematic Reviews and Meta-Analyses extension for Scoping Reviews (PRISMA-ScR) checklist and the recommendations of the Joanna Briggs Institute methodology for scoping reviews. The scoping review and subsequent systematic review will be completed by spring 2021.

**Results:**

The preliminary search has been conducted. After removing all duplicates, we found 2530 results. The review can now be advanced to the next steps of the methodology, including literature database queries with appropriate keywords, retrieval of the results in a reference management tool, and screening of titles and abstracts.

**Conclusions:**

The results of the search indicate that there is sufficient literature to complete the scoping review. Upon completion, the scoping review will provide a map of existing evidence on tele-ICU systems given the implementation context. Findings of this research could be used by researchers, clinicians, and implementation teams as they determine the appropriate setup of new or existing tele-ICU systems. The need for future research contributions and systematic reviews will be identified.

**International Registered Report Identifier (IRRID):**

DERR1-10.2196/19695

## Introduction

### Background

Since the first experiments in the late 1970s, telemedicine has increasingly been adopted in intensive care settings [1]. Recent figures indicate that telemedicine technologies are now in use for approximately 15% of intensive care beds in the United States [1-3]. Similar technologies have also been in use in Europe. An illustration of this trend is found at the *Charité—Universitätsmedizin Berlin*, a large university hospital in Germany, where an intensive care unit (ICU) telemedicine program focusing on quality improvement in postoperative care is being implemented [4].

An ICU is defined as a system for the provision of specialized medical and nursing care to patients located in a specific area of a hospital [5]. The term tele-ICU collectively refers to the telemedical systems that are deployed to extend or complement the capabilities of the ICU. Tele-ICU interventions are defined as the remote delivery of clinical intensive care services through conferencing and monitoring technologies [2,3,6]. Depending on the system setup, this may include audio-visual systems allowing two-way real-time communication between intensivists, bedside clinical staff, specialists, subspecialists, and patients [7]. This scoping review will focus on the implementation of these conferencing and monitoring technologies.

A range of rationales for implementing telemedicine technologies in intensive care has been suggested. Tele-ICU interventions have been described as a cost-effective response to a lack of intensive care availability. In the United States in particular, tele-ICUs have been used to address shortfalls in intensive care staffing, enabling intensivists to remotely monitor a large number of patients [6]. Additionally, tele-ICU technology allows access to populations in remote areas, thereby making specialty intensive care consultations more widely available [8]. Other applications have focused on increasing adherence to evidence-based best practices [3,9], using benchmark performance data [6]. Telemedicine in intensive care has been used as a way to improve patient safety by reducing alarm fatigue [9]. Applications in medical education, for instance, during the training of resident intensivists, has also been described [10].

Telemedicine in intensive care has been characterized by high variability in the modality and context of implementation. This is exemplified by the variety of technology setups found in the literature [6]. Tele-ICU systems may be organized according to numerous models regarding their system architecture, care intensity, and staffing pattern [7,11]. First, tele-ICU system architecture can be centralized (ie, “hub and spokes”) or decentralized (ie, distributed across multiple organizations) [3]. In both configurations, systems can connect multiple institutions across organizational boundaries (ie, different institutions) and, in some cases, in wide geographic areas (from local to international). Second, tele-ICU care processes can be characterized by their care intensity [1]. Higher-intensity models feature escalation protocols for staff response combined with a proactive clinical approach. Lower-intensity setups consist of discontinuous patient coverage combined with a reactive approach to patient events [12,13]. These two tele-ICU types of engagement protocols have also been respectively labeled as “direct intervention” and “monitoring and notify” [13]. Third, staffing patterns and care team composition vary across systems. Tele-ICUs accommodate different intensivist presence times at the bedside during the day, night, or weekend, based on the needs and resources of the ICU and tele-ICU units [14]. The wider care team composition also presents some differences between tele-ICUs. It may include nurses, pharmacists, and nonclinical staff.

More generally, tele-ICUs also reflect the various forms of ICU organization found across countries or regions with different standards of intensive care. ICUs in the United States are characterized by the dominance of the “open model,” with approximately 80% of ICUs staffed by nonintensivists. In contrast, in many countries, the “closed model” is predominant [10]. In this model, patients are systematically transferred to a trained intensivist. It follows that tele-ICUs have been integrated and adapted to ICUs with different models to fulfill different clinical and organizational needs.

### Literature Gap

Researchers have suggested that the setup characteristics of telemedicine systems play an important role in the success of tele-ICU implementation [15]. The context of implementation has been a determinant of the form of tele-ICU organization [16]. Implementation context is defined as the structures and processes inside which a technology is deployed [17]. The organizational context is a key aspect to consider when developing new tele-ICU systems and evaluating the effectiveness of telemedicine intensive care interventions.

Extensive literature has been produced on tele-ICU interventions, including several systematic reviews [18-23]. The main focus of these reviews has been on the benefits of telemedicine implementation with regard to clinical and economical outcomes. Most studies have employed semiexperimental research designs, which include before/after comparisons with or without a control group [24]. To date, three meta-analyses have been performed for tele-ICU with hospital mortality and length of stay as outcomes [24]. Other reviews in the domain involve additional outcomes including staff satisfaction, adherence to best practices, and rate of mechanical ventilation [9].

Based on the conclusions of these reviews, benefits derived from tele-ICU implementation appear heterogeneous [15,25]. A recent systematic review by Chen et al [23] identified a positive effect of tele-ICU with a reduction in ICU and hospital mortality. However, in other tele-ICU studies, benefits derived from using telemedicine technologies in intensive care settings could not be detected [15], while other studies pointed to mixed results with a reduction in ICU mortality but no relevant impact on in-hospital mortality [18]. The variability in outcomes highlights that the benefits derived from tele-ICU interventions depend on the organization where it is deployed [11] and how the technology is applied [26]. The choice of a relevant implementation model given its context is therefore an important aspect to achieve efficacy [24]. The need for additional research about technology characteristics and implementation context has been highlighted [17]. For instance, Kahn et al noted a lack of research contributions on the factors influencing organizational and clinical effectiveness [15]. Researchers have also noted that there are currently no recommended guidelines for determining the most appropriate tele-ICU setup or composition [6].

In recent years, scoping reviews have been employed to provide an overview of the field of literature and examine emerging evidence for new types of interventions [27]. This research method has become a valuable tool for providing evidence synthesis for complex systems. A scoping review may be used to efficiently access mapping of evidence and peer-reviewed literature for a range of outcomes and thus serve as a reference for teams involved in the implementation of tele-ICUs. Scoping reviews can also help evaluate research gaps and identify the need for future systematic reviews in specific subdomains [28]. We did not find an existing scoping review on the topic after a preliminary search of online databases.

### Aim

The purpose of this publication is to provide a comprehensive overview of telemedicine outcomes in relation to the ICU implementation context. We will map out evidence on outcomes of the use of telemedicine technology in intensive care and seek to offer perspectives on possible configurations of tele-ICU technologies, based on the implementation context and use case.

## Methods

### Research Team and Study Design

This protocol was developed using guidance from the methodological framework on scoping reviews by Arksey and O’Malley [28], and subsequent developments by the Joanna Briggs Institute [29]. This framework consists of a number of consecutive stages as follows: (1) identifying the research question, (2) identifying relevant studies, (3) selecting studies, (4) charting the data, and (5) collating, summarizing, and reporting results. We will use the Preferred Reporting Items for Systematic Reviews and Meta-Analyses extension for Scoping Reviews (PRISMA-ScR) checklist to report our results [30]. At present, the international Prospective Register of Systematic Reviews (PROSPERO) does not accept scoping review protocols for publication, so this protocol was not registered.

The research team consists of a doctoral researcher with a background in health economics (CG); a professor for digital health, who is a consultant anesthesiologist and a computer scientist (FB); a professor of medical informatics (MB); a consultant anesthesiologist with specialty in intensive medicine, who is a team coordinator for the intensive care telemedicine project (BW); a postdoctoral researcher in anesthesiology residency with a background in digital health (ASP); and a professor of information systems, digital transformation, and information technology infrastructure (DF).

### Step 1: Identifying the Research Question

The purpose of this scoping review is to map out findings and evidence about tele-ICU in light of its implementation context. The main research question for this review is as follows: what are the benefits of using telemedicine technology in intensive care? More specifically, the following subquestions are formulated: (1) Are there implementation contexts (eg, hospital type) that are more conducive to positive outcomes of telemedicine in intensive care? (2) What tele-ICU configurations (eg, staffing) are more appropriate for certain implementation contexts? (3) What range of outcomes exist for tele-ICU implementation in the literature and to what extent have these been extensively researched?

### Step 2: Identifying Relevant Studies

The databases Web of Science Core Collection, MEDLINE (via Web of Science, Clarivate Analytics), Library, Information Science & Technology Abstracts, ERIC, PsycINFO, PSYNDEX, and CINAHL (via EBSCO Host, EBSCO Information Services), as well as IEEE (via IEEE Xplore, Institute of Electrical and Electronics Engineers) have been searched for peer-reviewed literature. The search queries have been reviewed by both the information specialist and intensive care clinicians in the research team. The electronic database search will be supplemented by a manual search for grey literature. We have scanned the checklist of the Canadian Agency for Drugs and Technologies in Health to look for additional literature references.

We have followed the guidelines of the Peer Review of Electronic Search Strategies (PRESS) to formulate the queries. The exact search query used for Web of Sciences and EBSCO Host can be found in Multimedia Appendix 1. An overview of the search terms is shown in Table 1.

**Table 1:** Overview of the search terms.

Topic	Search keywords
Intensive care	ICU^a^ Intensive care unit Intensive care Acute care Critical care
Telemedicine	Tele-ICU Remote presence Virtual ICU eHealth mHealth^b^ Digital health Telemedicine Telecare Telehealth Digital intervention

^a^ICU: intensive care unit.

^b^mHealth: mobile health.

The search terms have been used in combination with the appropriate Boolean operators to formulate the search query. Search records, which include titles and abstracts, have been collated and managed using the reference management software Citavi version 6 (Swiss Academic Software). Duplicates have been identified and removed from the selection using Citavi duplicate management functionality.

A first selection of references will be performed based on screening of the titles and abstracts. Based on this selection, the full text will be retrieved and a detailed screening will be performed. The rationale for excluding studies on full-text screening will be documented and reported in the review. Full citations and a copy of the eligible studies will be retrieved and imported into Citavi.

Scoping reviews typically do not require to make a quality assessment of primary research. However, where applicable, we will complete a quality assessment of individual publications using adequate tools to appraise the quality of evidence.

### Step 3: Selecting Studies

A screening guide has been developed by the reviewers to lay out the inclusion and exclusion criteria. The selection process will be first conducted by a main reviewer (CG) and then validated by at least one reviewer in the research team. Divergence in classification will be resolved through discussion based on consensus of the reviewers. To ensure consistency in the selection of sources and the review methodology, a feasibility test will be conducted among the members of the research team with a sample of 100 publications from the preliminary search.

The study selection will be divided into two steps to include both secondary and primary literature. A secondary literature screen (“level I screen”) will seek to identify all secondary literature about telemedical technology used in ICUs. Publication titles and abstracts in the search results will be analyzed for inclusion. The criteria applied in the level I screen are as follows: (1) publication about telemedicine technology in intensive care, (2) research approach is a review of the primary literature, (3) no study design restriction (systematic reviews, simple reviews, and narrative reviews), (4) no country restriction, (5) language is English, German, Spanish, or French, (6) no date restriction (database will be searched from inception to present), and (7) publication in a peer-reviewed journal.

A primary literature screen (“level II screen”) will then be applied to identify relevant primary literature. Eligibility criteria in the level II screen are based on the PICO framework (“Patient Problem,” “Intervention,” “Comparison,” and “Outcome”) [31] and are structured as follows: (1) participant: patients admitted and medical staff working in the ICU; (2) intervention: implementation of telemedicine technology in the ICU; (3) comparison: intensive care delivered via telemedicine compared with standard of care or ICU without telemedicine technology; (4) outcome: all outcomes are accepted for inclusion, such as clinical outcomes, economic outcomes, staff and patient satisfaction, and guideline compliance. Publications solely based on expert opinion (ie, editorials) will therefore not be included in the review. Additionally, all study designs will be considered, including both qualitative and quantitative research.

Publications about the use of telemedicine for neonatal and pediatric ICUs (NICUs and PICUs, respectively) will not be included in this scoping review. The rationale for this exclusion is that the characteristics of the patient population and organization of NICUs and PICUs are greatly different from generalist ICUs and would be better addressed in a separate review.

### Step 4: Charting the Data

The purpose of step 4 is to determine the data points contained in the publications from the previous step. The data points necessary for the analysis will be tabulated in extraction sheets. The extraction sheets will then serve as a basis of the review work.

A list of data items will be selected based on the medical and technology expertise of the research team in the domains of intensive care and telemedicine. As Munn et al [27] noted, this process of charting relevant forms is by nature iterative and is expected to evolve as literature is reviewed. Data items will be charted for this review and will enable analysis of the implementation of tele-ICUs.

The extraction process will consist of collecting and codifying information contained in the publications that describe tele-ICU systems and their implementation context. As summarized in Table 2, context is defined according to the following five topics: (1) ICU clinical focus, (2) ICU type, (3) hospital type, (4) tele-ICU system configuration, and (5) implementation rationale. Tele-ICU configuration classification is determined on the basis of the following aspects: technical architecture, staff allocation, and mode of communication within the system.

Draft data charting forms will be developed and approved by the research team after independent pilot testing using a sample of publications (ie, 10 articles). Once consistent results are achieved and forms are approved, data from all included full-text articles will be charted by one member of the research team and verified by a second member to ensure all relevant data are charted.

**Table 2:** Overview of data extraction topics.

Topic	Description
1. ICU^a^ clinical focus	Level of specialization of the ICU. Example: Medical ICU or surgical ICU versus specialized ICU type (eg, neurological).
2. ICU type	Main organization model of the ICU. Example: Open ICU versus closed ICU.
3. Hospital type	Clinical setting where the tele-ICU is implemented. Example: Urban and tertiary hospital versus community and rural hospital.
4. Tele-ICU system configuration	Technical architecture, staff allocation, and mode of communication of the tele-ICU system. Example: A centralized system with a hub architecture providing intensive care expertise in real time versus a decentralized system with an open architecture providing scheduled care.
5. Implementation rationale	Main rationale given for implementing a tele-ICU system. Example: Extending ICU coverage versus improvement of care quality.

^a^ICU: intensive care unit.

### Step 5: Collating, Summarizing, and Reporting the Results

We will group the studies by the context of use and rationale for implementation. To synthesize results, we will form clusters of similar publications by classifying the data items collected. This method will allow us to analyze and compare evidence of tele-ICU implementation within each publication cluster.

We will present the results of the synthesis in the form of a series of tables, graphs, and visual representations.

## Results

**Figure 1:**
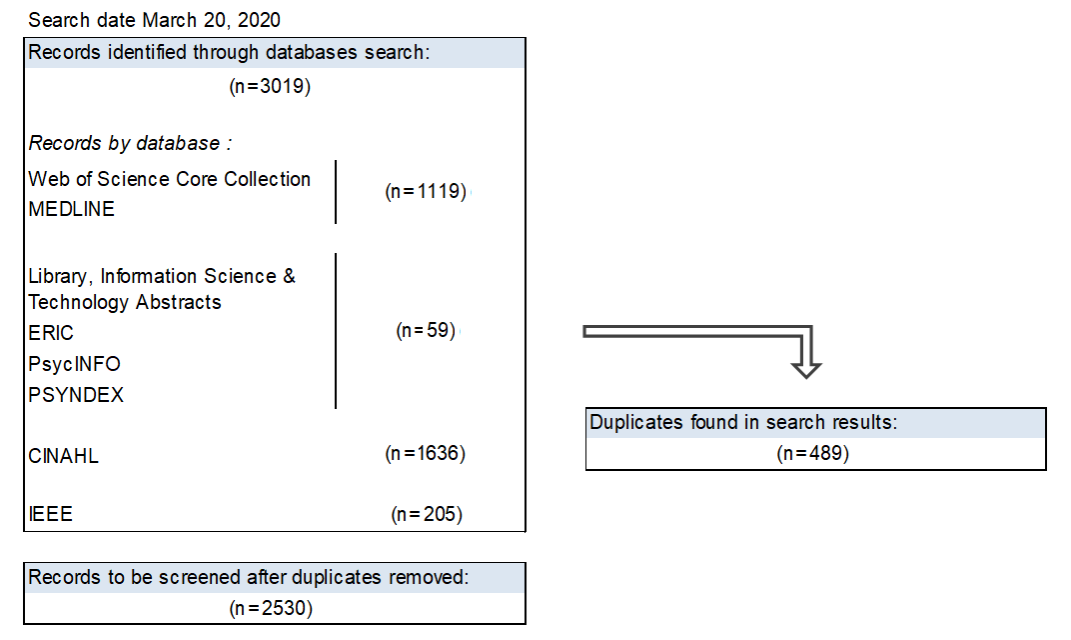
Literature search flow diagram.

A preliminary research was completed to assess existing literature and ensure that no other scoping review with the same focus has been published so far. The preliminary electronic database searches were carried out in March 2020. As described in step 2 of this protocol, research results from MEDLINE, IEEE, ERIC, PsycINFO, PSYNDEX, and CINAHL were downloaded. A total of 3019 results were retrieved, of which 489 were identified as duplicates. Figure 1 shows a flow diagram with the records identified through the database preliminary search. The remaining steps (3 to 5) of the scoping review will be completed by spring 2021.

## Discussion

### Preliminary Findings

The literature search yielded 2530 results after removing duplicates. The scoping review will provide a map of existing evidence on tele-ICU given the implementation context. The research findings could be used by researchers, clinicians, and implementation teams as they determine the appropriate setup for new or existing tele-ICU systems.

### Limitations

Some limitations can be identified in the research approach proposed in this protocol. First, this review will seek to synthesize evidence from publications that are using heterogeneous methodologies. This will pose a limit on the ability to draw generalization from the findings of this review. Second, the search terms and the study selection described in this protocol have been selected based on the expertise of the research team in the areas of anesthesiology, intensive care medicine, technology, and evidence research, as well as the existing literature, rather than according to pre-existing research frameworks or categories. This may represent a bias that the research team will need to consider when discussing the findings of the scoping review.

### Conclusions

We found that sufficient literature is available to complete the remaining steps of the methodology. To our knowledge, this is the first scoping review to examine the use of telemedicine in intensive care with a focus on the implementation context. Our research will contribute to the identification of where more evidence is needed to support the development of tele-ICU technology, with the appropriate configuration for its context and use case. The need for future research contributions and systematic reviews will be identified.

## Appendix

Multimedia Appendix 1 Search query.

## Abbreviations

ICU: intensive care unit
NICU: neonatal intensive care unit
PICU: pediatric intensive care unit
