# Supplementary material for: Telemedicine in Intensive Care Units: Protocol for a Scoping Review
Source: JMIR Res Protoc. 2020 Dec 31;9(12):e19695. doi: 10.2196/19695 (PMC7808887; doi:10.2196/19695)
Supplement: Multimedia Appendix 1 [file resprot_v9i12e19695_app1.docx]

Full Search Query

| **Search Engine** | **Query Text** |
| --- | --- |
| Web of Science Search | TOPIC: (  ("intensive care unit" OR  icu OR  “intensive care” OR  “acute care” OR  "critical care")  AND  (tele-ICU OR  teleICU OR  “tele ICU” OR  "Digital health" OR  e-health OR  telemedicine OR  telecare OR  telehealth OR  ehealth OR  mhealth OR  “remote presence” OR  “Virtual ICU”  "Digital Intervention")  ) |
| EBSCO Host | ("intensive care unit" OR  icu OR  “acute care” OR  "critical care")  AND  (tele-ICU OR  teleICU OR  “tele ICU” OR  "Digital health" OR  e-health OR  telemedicine OR  telecare OR  telehealth OR  ehealth OR  mhealth OR  "Digital Intervention")  ) |
| IEEE Xplore | ("intensive care unit" OR  icu OR  “acute care” OR  "critical care")  AND  (tele-ICU OR  teleICU OR  “tele ICU” OR  "Digital health" OR  e-health OR  telemedicine OR  telecare OR  telehealth OR  ehealth OR  mhealth OR  "Digital Intervention")  ) |
